# Conversion of Squid Pens to Chitosanases and Proteases via *Paenibacillus* sp. TKU042

**DOI:** 10.3390/md16030083

**Published:** 2018-03-08

**Authors:** Chien Thang Doan, Thi Ngoc Tran, Van Bon Nguyen, Anh Dzung Nguyen, San-Lang Wang

**Affiliations:** 1 Department of Chemistry, Tamkang University, New Taipei City 25137, Taiwan; doanthng@gmail.com (C.T.D.); tranngoctnu@gmail.com (T.N.T.); 2 Department of Science and Technology, Tay Nguyen University, Buon Ma Thuot City 630000, Vietnam; bondhtn@gmail.com; 3 Institute of Biotechnology and Environment, Tay Nguyen University, Buon Ma Thuot City 630000, Vietnam; nadzungtaynguyenuni@yahoo.com.vn; 4 Life Science Development Center, Tamkang University, New Taipei City 25137, Taiwan

**Keywords:** chitin, chitosan, squid pens, chitosanase, protease, α-glucosidase inhibitors, *Paenibacillus*

## Abstract

Chitosanases and proteases have received much attention due to their wide range of applications. Four kinds of chitinous materials, squid pens, shrimp heads, demineralized shrimp shells and demineralized crab shells, were used as the sole carbon and nitrogen (C/N) source to produce chitosanases, proteases and α-glucosidase inhibitors (αGI) by four different strains of *Paenibacillus*. Chitosanase productivity was highest in the culture supernatants using squid pens as the sole C/N source. The maximum chitosanase activity of fermented squid pens (0.759 U/mL) was compared to that of fermented shrimp heads (0.397 U/mL), demineralized shrimp shells (0.201 U/mL) and demineralized crab shells (0.216 U/mL). A squid pen concentration of 0.5% was suitable for chitosanase, protease and αGI production via *Paenibacillus* sp. TKU042. Multi-purification, including ethanol precipitation and column chromatography of Macro-Prep High S as well as Macro-Prep DEAE (diethylaminoethyl), led to the isolation of *Paenibacillus* sp. TKU042 chitosanase and protease with molecular weights of 70 and 35 kDa, respectively. For comparison, 16 chitinolytic bacteria, including strains of *Paenibacillus*, were investigated for the production of chitinase, exochitinase, chitosanase, protease and αGI using two kinds of chitinous sources.

## 1. Introduction

Chitin is one of the most abundant renewable natural polymers, second only to cellulose. Chitin and its derivatives, such as chitosan and chitin/chitosan oligomers, possess great economic value because of their diverse biological activities and biotechnological applications. Among chitin-containing bioresources, crab shells, shrimp shells and squid pens have the highest chitin content [1,2]. These marine byproducts contain not only chitin, but proteins and mineral salts as well. Chemical treatments, using strong alkali and acids for deproteinization and demineralization, are traditionally used to prepare chitin and chitosan from crab shells, shrimp shells and squid pens [2,3,4,5]. However, these chemical procedures have several drawbacks, including the large amount of protein-containing wastewater produced due to the high alkali content [1]. The development of green techniques, such as the application of enzymes for chitin extraction, is gaining greater attention. The cost of using enzymes can be decreased by incorporating deproteinization via fermentation, which can be achieved by proteolytic microorganisms [1,2,3,4,5,6].

Chitin and chitosan have been widely used as C/N (carbon/nitrogen) sources for the production of bacterial chitinolytic enzymes [1]. To reduce costs and recycle chitinous processing materials more efficiently, shrimp shells, crab shells and squid pens have been used as the sole C/N sources for screening chitinolytic and proteolytic enzyme-producing bacteria [7,8,9,10]. Unlike chitin and chitosan, chitin-containing fishery byproducts show greater potential as C/N sources since they can produce chitinolytic enzymes, proteases and other high-value-added bioactive materials [11,12,13,14,15,16,17,18,19].

Among chitin-containing fishery byproducts, squid pens contain the highest percentage of proteins and are most suitable for producing enzymes [7,8,9,10,11] and various bioactive compounds, such as antioxidants [13,14,15], α-glucosidase inhibitors (αGI) [16,17,18], biofertilizers [19,20], compounds with antitumor properties [13], biosurfactants [21], exopolysaccharides [21,22,23], insecticidal materials [24,25] and chitin, as well as chitin oligomers [20].

Many strains of *Paenibacillus* have applications in the agricultural, industrial and medical fields [26]. Recently, some strains of *Paenibacillus* were investigated for their ability to produce α-glucosidase inhibitors [16,17,18], exopolysaccharides [21,22,23] and anti-inflammatory antioxidants [14] when squid pens were the sole C/N source. However, few reports exist about chitinolytic and/or proteolytic enzymes from *Paenibacillus*, especially using squid pens. As such, there is interest in using squid pens to produce chitosanase and protease via fermentation with *Paenibacillus*.

In this study, four chitinous materials, including squid pen powder (SPP), demineralized shrimp shell powder (deSSP), shrimp head powder (SHP) and demineralized crab shell powder (deCSP), were used as the sole C/N sources for chitosanase and protease production by *Paenibacillus* sp. TKU042 and three other *Paenibacillus* strains. Optimal culture conditions and enzyme purification were investigated as well as the characterization of the *Paenibacillus* sp. TKU042 chitosanases and proteases. In previous studies, *Paenibacillus* sp. TKU042 has been reported to produce α-glucosidase inhibitors in a deCSP-containing medium. Therefore, a comparison of the α-glucosidase inhibitors produced by the four *Paenibacillus* strains was also performed.

## 2. Results and Discussion

### 2.1. Screening of Chitinous Materials as Sole C/N for Chitosanase Production

To explore the production of chitosanase and protease via *Paenibacillus*, four strains (TKU029, TKU032, TKU037, and TKU042) were incubated in a liquid medium containing 1% (*w*/*v*) of different chitinous materials, which were used as the sole C/N source. These included SPP, deSSP, SHP and deCSP. The *Paenibacillus* strains exhibited highest chitosanase activity in the culture supernatants of medium containing SPP, with a productivity of 0.928 U/mL for TKU042, 0.714 U/mL for TKU032, 0.529 U/mL for TK037 and 0.440 U/mL for TKU029. The incubation time to achieve maximum chitosanase activity was shortest for TKU042 (2 days) compared to the other strains (3 days). After two days of fermentation, *Paenibacillus* sp. TKU042 showed the highest chitosanase activity (0.928 U/mL) in medium containing SPP, compared to SHP (0.397 U/mL), deSSP (0.201 U/mL) and deCSP (0.216 U/mL).

Recently, *Paenibacillus* sp. TKU042 was suggested as a potential bacterium for the food and medical industries, thanks to its ability to produce αGI with high efficiency via fermentation in a commercial medium (nutrient broth), and especially on chitinous materials [16,17,18]. Therefore, this study not only screened the optimal chitinous materials (1% *w*/*v*) for producing chitosanase and protease, but also examined the production of α-glucosidase inhibitors. SPP was best for producing chitosanase (0.928 U/mL), while protease activity reached its maximum value (0.622 U/mL) in SHP-containing medium after 2 days of fermentation; higher αGI activity was found in both deSSP (182.55 U/mL) and deCSP (182.09 U/mL) mediums (after 2 days of fermentation). These results were consistent with the research of Nguyen et al. [17], which showed that *Paenibacillus* sp. TKU042 produced higher αGI activity in deCSP- and deSSP-containing media.

With its high ratio of proteins and low ratio of mineral salts, SPP is suitable for producing chitosanase via many bacterial strains [1,2,10,15]. In this study, SPP was also found to be the best C/N source for chitosanase production by *Paenibacillus* sp. TKU042.

### 2.2. Effect of SPP Concentration on Chitosanase, Protease and αGI Production

**Figure 1:**
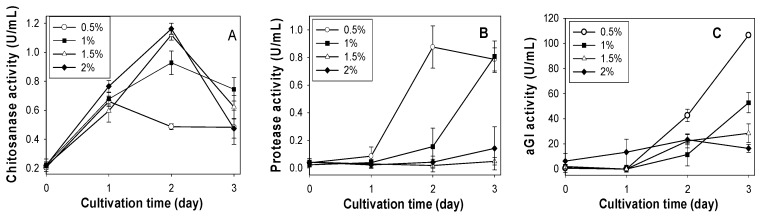
Effect of SPP concentration on the production of chitosanase, protease and αGI by *Paenibacillus* sp. TKU042. (**A**) chitosanase activity; (**B**) protease activity; (**C**) αGI activity. *Paenibacillus* sp. TKU042 has been reported to produce higher αGI activity in deCSP than any of the other three chitinous materials (SPP, deSSP and SHP) when used as the sole C/N source [17]. To examine whether the production of chitosanase (1.5% SPP) and protease (0.5% SPP) may be enhanced by a combination of deCSP and SPP, 1% deCSP was added to the medium. As seen in Figure 2, the combination of SPP with deCSP enhanced the production of protease (Figure 2B), but not chitosanase (Figure 2A) or αGI (Figure 2C).

According to the above results, SPP was considered the best C/N source for chitosanase production by *Paenibacillus* sp. TKU042. To determine the optimal SPP concentration for chitosanase, protease and αGI production, the basal medium (0.1% K_2_HPO_4_, 0.05% MgSO_4_·7H_2_O) was supplemented with different concentrations (0.5%, 1%, 1.5%, 2%). As shown in Figure 1A, the highest chitosanase activity was found for 1.5% SPP (1.120 U/mL, 2 days) and 2% SPP (1.162 U/mL, 2 days). Conversely, a lower concentration of SPP (0.5%) gave better results for the production of protease (0.876 U/mL, 2 days) and αGI (106.66 U/mL, 3 days). Overall, higher concentrations (2% or 1.5%) of SPP are suitable for chitosanase production, but not for protease or αGI. Due to there being no significant differences in chitosanase activity between 1.5% (1.120 U/mL) and 2.0% SPP (1.162 U/mL), 1.5% was chosen as the most suitable concentration for chitosanase production.

### 2.3. Production of Chitosanase, Protease and αGI from SPP and deCSP by Different Bacteria

To make a wide comparison between the production of chitosanase, protease and αGI by different *Paenibacillus* strains, 12 chitinolytic bacteria strains isolated from Taiwanese soils were tested. SPP (suitable for chitosanase production) and deCSP (suitable for αGI production) were used as the sole C/N sources. Since the substrates used for analyzing exochitinase (*p*-nitrophenyl-*N*-acetyl-β-d-glucosaminide) and chitinase (colloidal chitin) were similar to that for chitosanase (water-soluble chitosan), the activities of exochitinase and chitinase were also examined and compared.

As shown in Table 1, the highest protease activity was found in four *Paenibacillus* strains: TKU032 (1.366 U/mL), TKU042 (1.257 U/mL), TKU037 (1.203 U/mL) and TKU029 (0.925 U/mL). For αGI production, *Paenibacillus* strains TKU042 (185.45 U/mL), TKU037 (180.77 U/mL) and TKU 032 (178.38 U/mL) again produced the highest results; TKU029 (62.90 U/mL) was the only exception. For chitinase production, *Paenibacillus* sp. TKU042 (0.182 U/mL) showed higher production than TKU037 (0.135 U/mL), TKU029 (0.118 U/mL) or TKU032 (0.072 U/mL). *Serratia ureilytica* TKU013 produced the most exochitinase (0.276 U/mL) and chitinase (0.266 U/mL). No significant differences were found between the 16 strains for chitosanase production. Among the four *Paenibacillus* strains, the highest chitosanase activity (0.122 U/mL) was found in *Paenibacillus macerans* TKU029. Based on these results, deCSP was a better C/N source for the production of αGI and protease by *Paenibacillus*, especially *Paenibacillus* sp. TKU042, which generated higher chitinase, chitosanase, protease and αGI activities.

When 1% SPP was used as the sole C/N source (Table 2), *Paenibacillus* strains showed higher chitosanase and αGI activity than the other genus strains. Highest chitosanase activity was found in *Paenibacillus* sp. TKU042 (0.928 U/mL), followed by *Paenibacillus macerans* TKU029 (0.857 U/mL). αGI production was highest in *Paenibacillus* strains TKU037 (175.80 U/mL), TKU032 (175.40 U/mL), TKU042 (88.30 U/mL) and TKU029 (60.13 U/mL). *Serratia ureilytica* TKU013 generated the highest protease (0.910 U/mL) and exochitinase (11.545 U/mL) activity.

*Paenibacillus* strains TKU042, TKU037, TKU032 and TKU029 demonstrated higher αGI activity in deCSP (Table 1) than SPP (Table 2). These results were consistent with previous reports [17]. This study also found two strains of *Bacillus* (TKU004 and TKU038) with the potential to produce αGI. *B. licheniformis* TKU004 produced 174.59 U/mL αGI in SPP (Table 2) and *B. mycoildes* TKU038 produced 111.67 U/mL αGI in deCSP (Table 1). Similar results were also found in *Bacillus subtilis* B2 [27] and *B. subtilis* MORI [28], which were cultured for αGI production in okara and soybean extracts, respectively.

Recently, many strains of *Paenibacillus* have been found to produce exopolysaccharides when cultured in medium containing squid pens [22,23,29] or carbohydrates, such as sucrose [30]. Interestingly, a strain of *Bacillus licheniformis* (OSTK95) also produced exopolysaccharides in glucose-containing medium [31]. In fact, *Paenibacillus* was originally included within the *Bacillus* genus and was then reclassified as a separate genus in 1993 [32]. With results similar to *Paenibacillus*, the fact that *Bacillus licheniformis* TKU004 produces αGI (Table 2) and *B. licheniformis* OSTK95 produces exopolysaccharides [31] suggests that *Bacillus* strains, especially *B. licheniformis*, could potentially be developed for the production of αGI and exopolysaccharides.

In this study, all bacterial strains were isolated using chitin- and protein-containing media as the sole C/N sources. It was assumed that to obtain nutrition, the bacteria would produce chitinolytic enzymes or proteases by hydrolyzing the chitin or protein in the medium. For this reason, the chitinolytic enzymes, including exochitinase and chitinase, were also studied. As shown in Table 1 and Table 2, most of the tested strains showed chitinase activity on both SPP and deCSP. Among all the bacteria, *Paenibacillus* sp. TKU029 produced the highest chitinase activity on SPP (0.376 U/mL).

Extracellular chitinases were divided into two types: exochitinase or endochitinase. While endochitinase activity was defined as random cleavage at internal points in the chitin chain, exochitinase activity was defined as a progressive action starting at the non-reducing end of chitin, with the release of chitobiose or *N*-acetylglucosamine units [9]. In this study, chitinase was produced by most of the bacteria, but exochitinase was only found in four strains: *Bacillus licheniformis* TKU004, *Serratia marcescens* TKU011, *Serratia ureilytica* TKU013 and *Serratia* sp. TKU020. None of the *Paenibacillus* strains produced exochitinase (Table 1 and Table 2). Therefore, *Paenibacillus* TKU042 chitinase is classified as an endochitinase.

### 2.4. Purification and Characterization of Chitosanase and Protease

**Figure 3:**
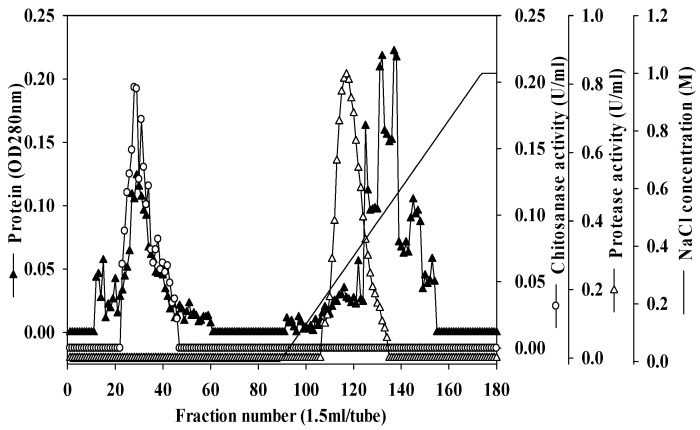
Elution profile of chitosanase and protease on Macro-Prep High S.

Unlike αGI, there are few reports of chitinolytic enzymes and proteases being produced by *Paenibacillus* species. Chitosanase produced by *Paenibacillus ehimensis* NRRL B-23118 has been studied, but purification was not performed [33]. To compare the different chitinases and proteases produced by *Paenibacillus* sp. TKU042 against other reported strains, the chitosanase and protease of *Paenibacillus* sp. TKU042 were purified from the culture supernatant by a series of steps. The enzymes were eluted by Macro-Prep High S chromatography with a linear gradient of 0–1 M NaCl, using the same buffer (Figure 3). The eluted peak fractions were pooled for further purification. After two rounds of chromatography (data not shown), approximately 0.52 mg of chitosanase and 0.20 mg of protease were obtained (Table 3 and Table 4). The specific activity and recovery yields of chitosanase were 3.09 U/mg and 3.09%, respectively (Table 3), while for protease they were 38.19 U/mg and 8.70%, respectively (Table 4). 

**Figure 4:**
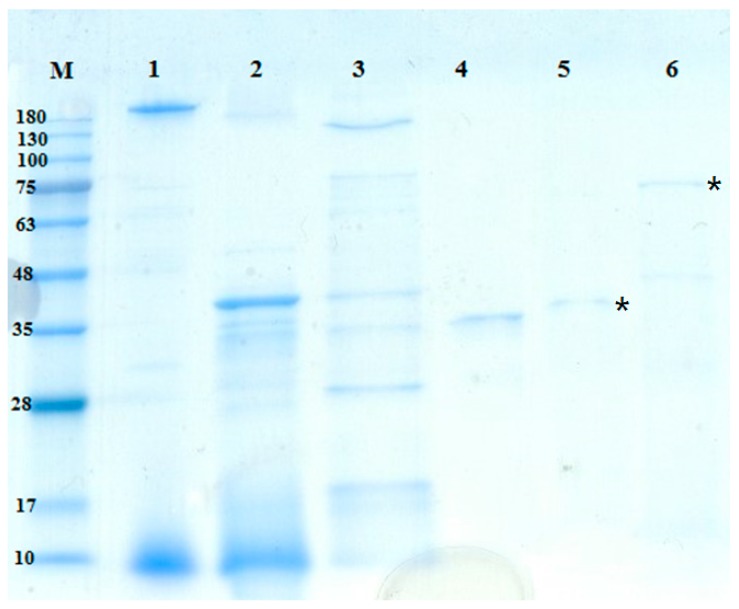
SDS-PAGE analysis of the protease and chitosanase produced by TKU042. Lanes: M. molecular markers; 1. cultural supernatant; 2. crude enzyme after precipitation of ethanol; 3. adsorbed chitosanase after Macro-Prep High S Chromatography; 4. adsorbed protease after Macro-Prep High S Chromatography; 5. purified protease after Sephacryl 200 Chromatography; 6. purified chitosanase after Macro-Prep DEAE Cartridge Chromatography; *, the purified enzymes.

The molecular masses of the TKU042 chitosanase and protease were approximately 70 kDa and 35 kDa, as determined by the SDS-PAGE (sodium dodecyl sulfate polyacrylamide gel electrophoresis) (Figure 4). The molecular weight of TKU042 chitosanase (70 kDa) was larger than that of *B. subtilis* KH1 (28 kDa) [34], *B. ehimensis* EAG1 (31 kDa) [35], *B. subtilis* IMR-NK1 (36 kDa) [36], *B. licheniformis* A10 (40.5 KDa) [37], *B. megaterium* p1 (22, 39.5, 43 kDa) [38], *B. cereus* S1 (45 kDa) [39], *Bacillus* sp. KCTC0377BP (45 kDa) [40], *Bacillus* sp. P16 (45 kDa) [41], *B. mycoides* TKU038 (48 kDa) [10], *Bacillus* sp. MET1299 (52 kDa) [42] and *Paenibacillus* sp. 1794 (40 kDa) [43]; only *Paenibacillus fukuinensis* D2 (67 kDa) had a similar weight [44]. Meanwhile, *Paenibacillus* sp. TKU042 protease (35 kDa) fell within the recorded range (19.95–67.61 kDa) from other strains of *Paenibacillus* [45,46] and *Bacillus* [47,48,49].

## 3. Materials and Methods

### 3.1. Materials

Squid pens, crab shells and shrimp shells were obtained from Shin-Ma Frozen Food Co. (I-Lan, Taiwan) [17]. Shrimp head powder was obtained from Fwu-Sow Industry (Taichun, Taiwan). Demineralized crab shells and demineralized shrimp shells were preparative via acid treatment [17]. *Saccharomyces cerevisiae* α-glucosidase, the substrates for determining enzyme activities (water-soluble chitosan, chitin (from shrimp shells) and *p*-nitrophenyl-*N*-acetyl-β-d-glucosaminide), the reference compounds (*p*-nitrophenol, tyrosine, *N*-acetylglucosamine and glucosamine) and the reagents (3,5-dinitrosalicylic acid and Folin-Ciocalteu) were all purchased from Sigma-Aldrich Corp. (Singapore). Sephacryl S200 was purchased from GE healthcare UK Ltd. (Little Chalfont, Buckinghamshire, England, UK). Macro-prep DEAE and Macro-Prep High S were obtained from Bio-Rad (Hercules, CA, USA). All other reagents were of the highest grade available.

### 3.2. Measurement of Enzyme Activities

#### 3.2.1. Chitosanase Activity

The measurement of chitosanase activity was performed according to a previously described method [10], with modifications. Water-soluble chitosan (1% in 50 mM phosphate buffer) was used as the substrate. The reaction was performed with 0.2 mL substrate and 0.2 mL enzyme solution and kept at 37 °C for 60 min. The amount of reducing sugar produced in the supernatant was determined by DNS reagent, with glucosamine as the reference compound. One unit of enzyme activity was defined as the amount of enzyme that produced 1 µmol of reducing sugar per min [10].

#### 3.2.2. Protease Activity

To measure protease activity, the enzyme solution (0.1 mL) was mixed with 0.1 mL substrate (1% casein in 50 mM phosphate buffer) and incubated for 60 min at 37 °C. The reaction was stopped by adding 0.6 mL TCA solution (5%). The reaction mixture was then measured as per the methods of Todd, with tyrosine as the reference compound [19]. One unit of protease activity was defined as the amount of enzyme required to release 1 µmol of tyrosine per min.

#### 3.2.3. Chitinase Activity

Measurement of chitinase activity was performed according to the methods described above for chitosanase, with slight modifications. Instead of water-soluble chitosan, colloidal chitin (1% in 50 mM phosphate buffer) was used as the substrate and *N*-acetylglucosamine was used as the reference compound instead of *N*-glucosamine [7].

#### 3.2.4. Exochitinase Activity

Exochitinase activity was determined as per the previously described methods [7], with modifications. The culture supernatant (50 µL) was mixed with 100 µL of *p*-nitrophenyl-*N*-acetyl-β-d-glucosaminide solution (1 g/L) and 500 µL of 50 mM sodium acetate buffer (pH 4.6), then incubated at 37 °C for 30 min. The color of *p*-nitrophenol (pNP) appeared once 325 µL of sodium carbonate (50 mM, pH 10.7) was added to the mixture solution. The final solution was measured at 410 nm. pNP was used as the reference compound. One unit of enzyme activity was defined as the amount of enzyme that produced 1 µM of pNP per min.

### 3.3. Measurement of Alpha Glucosidase Inhibitor

To measure αGI activity, the inhibitor solution (10 µL) was mixed with 10 µL of α-glucosidase solution (1 U) and 0.1 mL phosphate buffer (100 mM), then kept at 37 °C for 30 min [17]. 10 µL of substrate *p*-nitrophenyl glucopyranoside (pNPG) was then added to the mixture before incubation at 37 °C for 30 min. The reaction was stopped by adding 0.13 mL Na_2_CO_3_ solution (1 M). The final mixture solution was measured at 410 nm. The inhibition was calculated using the following formula,

Inhibition (%) = (A − B)/A × 100,

where A is the absorbance of the reaction blank at 410 nm (no inhibitor/sample), and B is the absorbance of the reaction at 410 nm in the presence of the inhibitor/sample. The concentration of an inhibitor that could inhibit 50% of enzymatic activity under assay conditions was defined as the IC_50_ value.

### 3.4. Screening of Chitinous Materials as Sole C/N for Enzyme Activity

Various kinds of chitin-containing materials, such as demineralized crab shell powder (deCSP), squid pen powder (SPP), shrimp head powder (SHP) and demineralized shrimp shell powder (deSSP) (*w*/*v*) were used as the sole sources of C/N at a concentration of 1%. Four *Paenibacillus* strains were grown in 100 mL of liquid medium in 250 mL Erlenmeyer flasks containing 1% of each chitinous material, 0.1% K_2_HPO_4_ and 0.05% MgSO_4_·7H_2_O. One mL of the seed culture was transferred into 100 mL of the medium and incubated for 3 days at 37 °C on a shaking incubator (150 rpm). Every 24 h, the culture broth was centrifuged (12,000× *g* at 4 °C for 20 min) and the supernatant was used for further measurements.

### 3.5. Effect of SPP Concentration on Enzymes and αGI Activity

*Paenibacillus sp.* TKU042 was grown in a 250 mL Erlenmeyer flask with 100 mL of basal medium (0.1% K_2_HPO_4_, and 0.05% MgSO_4_·7H_2_O) and a range of SPP concentrations (0.5–2%) at 37 °C on a shaking incubator (150 rpm). For subsequent experiments, the optimal SPP concentration was mixed with 1% deCSP, and the mixture of chitinous material and basal medium was cultured under similar incubation conditions. Every 24 h, the culture broth was centrifuged (12,000× *g* at 4 °C for 20 min), and the supernatant was used for further measurements.

### 3.6. Production of Enzymes and αGI from SPP and deCSP Using Different Bacteria

Sixteen chitolytic bacterial strains were grown on two kinds of chitinous materials (SPP and deCSP). The incubation supernatants from the 4th day were used to measure exochitinase, chitinase, chitosanase, protease and αGI.

### 3.7. Purification of Chitosanase and Protease

300 mL of cold ethanol (−20 °C) was added to 100 mL of culture supernatant and kept at 4 °C overnight for protein precipitation. The precipitate was collected by centrifugation at 12,000× *g* for 30 min and then dissolved in a small amount of 20 mM Tris buffer (pH 7) and dialyzed against the buffer. The resulting crude enzyme was loaded onto a Macro-Prep High S column that had been equilibrated with 20 mM Tris buffer. A gradient of NaCl (0–1 M) in the same buffer was applied to the eluted enzymes. The enzyme solutions were then chromatographed on a Macro-prep DEAE column or on a Sephacryl S200 column (1 cm × 100 cm). The molecular masses of the enzymes were determined using the SDS-PAGE methods.

## 4. Conclusions

*Paenibacillus* produces a variety of biofertilizers, antimicrobials, enzymes and exopolysaccharides with applications in agriculture, medicine, process manufacturing and bioremediation, some of which have already been commercialized [26]. Further study will reveal other means by which *Paenibacillus* can contribute to health and sustainable processes. In this study, the use of squid pens as the sole C/N source gave the best results for chitosanase production by *Paenibacillus* sp. TKU042. The molecular weight of the purified TKU042 chitosanase (70 kDa) was higher than the other *Paenibacillus* strains. Among the sixteen tested chitinolytic bacteria, *Paenibacillus* sp. TKU042 has the best potential to produce chitinolytic enzymes, protease and α-glucosidase inhibitors using squid pens as the sole C/N source.

## Figures and Tables

**Figure 2:**
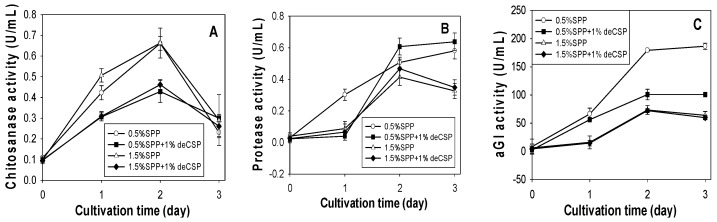
Effect of combining SPP with deCSP on the production of chitosanase, protease and αGI by *Paenibacillus* sp. TKU042. (**A**) chitosanase activity; (**B**) protease activity; (**C**) αGI activity.

**Table 1:** Comparison of chitinolytic enzyme, protease and αGI production by various bacteria using deCSP as C/N source.

Strain	Activity (U/mL)				
	Exochitinase	Chitinase	Chitosanase	Protease	aGI
*Paenibacillus macerans* TKU029	-	0.118 + 0.027	0.122 + 0.035	0.925 + 0.032	62.90 + 6.755
*P. mucilaginosus* TKU032	-	0.072 + 0.016	0.086 + 0.021	1.366 + 0.045	178.38 + 10.312
*Paenibacillus* sp. TKU037	-	0.135 + 0.018	0.069 + 0.019	1.203 + 0.041	180.77 + 11.620
*Paenibacillus* sp. TKU042	-	0.182 + 0.036	0.088 + 0.015	1.257 + 0.038	185.45 + 3.085
*Bacillus licheniformis* TKU004	-	-	0.089 + 0.022	0.121 + 0.023	91.29 + 8.692
*Bacillus subtilis* TKU007	-	0.163 + 0.023	-	0.178 + 0.011	96.06 + 7.008
*Lactobacillus paracasei* TKU010	-	0.216 + 0.033	-	0.113 + 0.009	-
*Serratia marcescens* TKU011	-	0.201 + 0.029	0.149 + 0.021	0.115 + 0.012	73.63 + 4.090
*Serratia ureilytica* TKU013	0.276 + 0.012	0.266 + 0.018	0.099 + 0.017	0.202 + 0.024	64.52 + 3.474
*Pseudomonas tamsuii* TKU015	-	0.193 + 0.030	0.105 + 0.013	0.103 + 0.008	51.54 + 4.627
*Serratia* sp. TKU016	0.152 + 0.021	0.104 + 0.012	0.110 + 0.022	0.101 + 0.007	68.962.292
*Serratia* sp. TKU020	0.121 + 0.016	0.102 + 0.015	0.151 + 0.013	0.099 + 0.009	73.63 + 6.420
*Bacillus cereus* TKU028	-	0.127 + 0.018	0.111 + 0.018	0.776 + 0.053	68.72 + 5.143
*Bacillus mycoildes* TKU038	-	-	0.101 + 0.017	0.463 + 0.036	111.67 + 1.036
*Bacillus mycoildes* TKU040	-	0.191 + 0.020	-	-	-
*Rhizobium* sp.TKU041	-	-	-	-	-

deCSP was used at the sole C/N source with a concentration of 1% (*w*/*v*). Culture conditions were set at 37 °C, 150 rpm shaking speed, 100/250 mL (volume of medium/flask) for two days. The cultures of fermented deCSP were obtained and centrifuged at 12,000× *g* for 20 min to collect the culture supernatants. (-) expressed activity lower than 0.1 U/mL and 10 U/mL for exochitinase and αGI, respectively.

**Table 2:** Comparison of chitinolytic enzyme, protease and αGI production by various bacteria using SPP as C/N source.

Strain	Activity (U/mL)				
	Exochitinase	Chitinase	Chitosanase	Protease	aGI
*Paenibacillus macerans* TKU029	-	0.376 + 0.026	0.857 + 0.015	0.135 ± 0.011	60.13 ± 6.844
*P. mucilaginosus* TKU032	-	0.101 + 0.017	0.155 + 0.016	0.193 ± 0.005	175.40 ± 0.606
*Paenibacillus* sp. TKU037	-	0.105 + 0.018	0.247 + 0.014	0.222 ± 0.004	175.80 ± 0.851
*Paenibacillus* sp.TKU042	-	0.185 + 0.019	0.928 + 0.014	0.199 + 0.005	88.30 ± 4.502
*Bacillus licheniformis* TKU004	1.211 + 0.017	0.074 + 0.017	0.120 + 0.014	0.707 + 0.020	174.59 ± 4.295
*Bacillus subtilis* TKU007	-	0.118 + 0.017	0.314 + 0.017	0.219 + 0.005	58.64 ± 7.368
*Lactobacillus paracasei* TKU010	-	-	0.166 + 0.015	0.090 + 0.010	-
*Serratia marcescens* TKU011	8.039 + 0.296	0.177 + 0.018	0.150 + 0.013	0.477 + 0.010	-
*Serratia ureilytica* TKU013	11.545 + 0.431	0.319 + 0.019	0.112 + 0.013	0.910 + 0.031	52.60 ± 8.418
*Pseudomonas tamsuii* TKU015	-	0.105 + 0.015	0.056 + 0.013	0.113 + 0.009	-
*Serratia* sp. TKU016	-	0.111 + 0.018	0.302 + 0.012	0.370 + 0.012	-
*Serratia* sp. TKU020	9.327 + 0.325	0.201 + 0.018	0.118 + 0.014	0.478 + 0.021	52.49 ± 0.778
*Bacillus cereus* TKU028	-	0.223 + 0.018	0.520 + 0.015	0.153 + 0.007	68.64 ± 3.972
*Bacillus mycoildes* TKU038	-	0.332 + 0.019	0.611 + 0.016	0.240 + 0.004	71.46 ± 3.635
*Bacillus mycoildes* TKU040	-	0.350 + 0.016	0.165 + 0.013	0.083 + 0.011	-
*Rhizobium* sp.TKU041	-	-	-	0.198 + 0.012	-

SPP was used at the sole C/N source with a concentration of 1% (*w*/*v*). Culture conditions were set at 37 °C, 150 rpm shaking speed, 100/250 mL (volume of medium/flask) for two days. The cultures of fermented deCSP were obtained and centrifuged at 12,000× *g* for 20 min to collect the culture supernatants. (-) expressed activity lower than 0.1 U/mL and 10 U/mL for exochitinase and αGI, respectively.

**Table 3:** Purification of the chitosanase from *Paenibacillus* sp. TKU042.

Step	Total Protein (mg)	Total Activity (U)	Specific Activity (U/mg)	Recovery (%)	Purification (Fold)
**Cultural supernatant**	211.43	53.03	0.25	100.00%	1.00
**Ethanol precipitation**	56.95	26.81	0.47	50.56%	1.88
**Macro-Prep High S**	4.59	4.62	1.01	8.71%	4.01
**Macro-Prep DEAE**	0.52	1.64	3.14	3.09%	12.51

*Paenibacillus* sp. TKU042 was cultured in 100 mL of liquid medium in a 250 mL Erlenmeyer flask containing 1% SPP, 0.1% K_2_HPO_4_ and 0.05% MgSO_4_·7H_2_O in a shaking incubator for 2 days at 37 °C. Protein content was estimated by a previously described method [10] using bovine serum albumin as the standard.

**Table 4:** Purification of the protease from *Paenibacillus* sp. TKU042.

Step	Total Protein (mg)	Total Activity (U)	Specific Activity (U/mg)	Recovery (%)	Purification (Fold)
**Cultural supernatant**	211.43	86.90	0.41	100.00%	1.00
**Ethanol precipitation**	56.95	60.35	1.06	69.45%	2.58
**Macro-Prep High S**	2.72	17.10	6.29	19.68%	15.30
**Sephacryl 200**	0.20	7.56	38.19	8.70%	92.91

*Paenibacillus* sp. TKU042 was cultured in 100 mL of liquid medium in a 250 mL Erlenmeyer flask containing 1% SPP, 0.1% K_2_HPO_4_ and 0.05% MgSO_4_·7H_2_O in a shaking incubator for 2 days at 37 °C. Protein content was estimated by a previously described method [10] using bovine serum albumin as the standard.
